# Host Genetic Diversity and Infectious Diseases. Focus on Wild Boar, Red Deer and Tuberculosis

**DOI:** 10.3390/ani11061630

**Published:** 2021-05-31

**Authors:** Javier Pérez-González, Juan Carranza, Remigio Martínez, José Manuel Benítez-Medina

**Affiliations:** 1 Biology and Ethology Unit, Veterinary Faculty, University of Extremadura, 10003 Cáceres, Spain; 2 Wildlife Research Unit (UIRCP), University of Córdoba, 14071 Córdoba, Spain; jcarranza@uco.es; 3 Infectious Pathology Unit, Veterinary Faculty, University of Extremadura, 10003 Cáceres, Spain; remimar@unex.es (R.M.); jmbenimed@unex.es (J.M.B.-M.)

**Keywords:** genetic diversity, disease spread, wild boar, *Sus scrofa*, red deer, *Cervus elaphus*, tuberculosis

## Abstract

**Simple Summary:**

Genetic diversity in wildlife is a matter of growing concern in contexts related to disease transmission and human health. Tuberculosis is a zoonotic disease with relevant consequences and can present high prevalence in wild boar and red deer populations. Here, we review studies on the genetic diversity of ungulates, wild boar, and red deer, and assess to what extent these studies consider its importance in the spread of disease. The relationship between host genetic diversity and the probability of disease spread is illustrated in Spanish populations of wild boar and red deer.

**Abstract:**

Host genetic diversity tends to limit disease spread in nature and buffers populations against epidemics. Genetic diversity in wildlife is expected to receive increasing attention in contexts related to disease transmission and human health. Ungulates such as wild boar (*Sus scrofa*) and red deer (*Cervus elaphus*) are important zoonotic hosts that can be precursors to disease emergence and spread in humans. Tuberculosis is a zoonotic disease with relevant consequences and can present high prevalence in wild boar and red deer populations. Here, we review studies on the genetic diversity of ungulates and determine to what extent these studies consider its importance on the spread of disease. This assessment also focused on wild boar, red deer, and tuberculosis. We found a disconnection between studies treating genetic diversity and those dealing with infectious diseases. Contrarily, genetic diversity studies in ungulates are mainly concerned with conservation. Despite the existing disconnection between studies on genetic diversity and studies on disease emergence and spread, the knowledge gathered in each discipline can be applied to the other. The bidirectional applications are illustrated in wild boar and red deer populations from Spain, where TB is an important threat for wildlife, livestock, and humans.

## 1. Introduction

Genetic diversity favors population conservation and individual survival. At population level, loss of genetic variation compromises evolutionary response to environmental change [1,2,3]. At the individual level, inbreeding reduces fitness [4,5]. Loss of population genetic diversity and inbreeding depression have received great attention in wildlife conservation and captive breeding programs [6,7,8,9,10]. However, genetic diversity of wildlife may be in growing concern in contexts related to disease transmission and human health.

Most human infectious diseases originate from animals [11,12,13,14]. Scientists have pointed out the significant threat of infectious diseases to global health, global economy, and global security [15,16,17]. Since 2020, humanity is becoming aware of the global effects of infectious diseases [18,19,20] and studies propose that the frequency of this threat is on the rise [12,21]. Efforts to minimize the emerging of infectious diseases are expected to increase. Research focusing on different aspects of disease emergence and transmission will support these efforts. 

A positive relationship between host genetic diversity of genetic markers and pathogen resistance (heterozygosity–fitness correlation) has been detected in different species [22,23,24,25,26,27,28,29,30,31,32]. Three hypotheses might explain this relationship [33]: the direct effect hypothesis, posing a direct link of the assessed genetic markers with parasite resistance, the local effect hypothesis, asserting that the assessed genetic markers are in linkage disequilibrium with fitness-linked loci, and the general effect hypothesis, claiming that genome-wide diverse individuals are relatively more resistant to diseases. Recent studies have shown that pathogen resistance is mainly achieved thanks to diversity of genes related to the immune system rather than genome-wide diversity [34,35], these results provide support to direct effect or local effect hypotheses. 

Studies on the relationship between diversity of immune genes and pathogen resistance have focused on the major histocompatibility complex (MHC). MHC genes drive the adaptive immune response, and their diversity promotes the number of pathogens recognized [35,36]. Diversity at MHC genes is maintained by balancing selection [37,38,39], and three mechanisms have been proposed to explain the advantage of MHC variability: overdominance or heterozygote advantage, rare allele advantage, and fluctuating selection [40]. 

Despite the fact that MHC genes have been the focus of considerable research [41,42], they only represent a fraction of the immune system. Other candidate genes have been shown to induce the relationship between genetic diversity and pathogen resistance or tolerance in hosts. For instance, Turner et al. [43] found that genetic diversity of cytokines is associated with variation in resistance to multiple pathogens, in a population of field voles (*Microtus agrestis*). Amino acid variation in the prion protein gene has been related to the probability of infection with chronic wasting disease and its progression following infection [44,45]. Quéméré et al. [22] have recently shown that diversity in Toll-like receptor genes in Alpine ibex (*Capra ibex*) affect *Brucella* infection status. 

Due to its relationship with pathogen resistance, host genetic diversity reduces pathogen prevalence, rate of pathogen adaptation to host, and pathogen virulence [46,47,48,49,50,51]. Therefore, host genetic diversity tends to limit disease spread in nature and buffers populations against epidemics [52,53,54,55]. Accordingly, genetic diversity of host populations deserves an increasing interest in contexts related to disease transmission and human health.

Patterns of genetic diversity have been broadly studied in population conservation contexts [6,8,56,57,58,59]. In addition to describing the patterns, these studies tend to investigate the factors and processes potentially affecting genetic diversity. Gene flow and genetic drift have been proposed as major processes affecting population genetic diversity [60,61,62,63,64]. On the other hand, past demographic history can also have a deep impact on the relationship between genetic diversity and fitness [65,66]. A sudden bottleneck can reduce genetic diversity and increase inbreeding and, hence, it tends to enhance the susceptibility to infectious diseases [67,68]. Contrarily, slow, long-term declines favor the action of natural selection that can purge deleterious alleles and favor population viability [69,70,71]. Therefore, a reduction in genetic diversity might have different outcomes over fitness of individuals and population viability. Nonetheless, the knowledge of the action of processes affecting genetic diversity can be important, not only for population conservation, but also to predict or manage the spread of infectious diseases. Wildlife management policies that reduce the risk of disease spread might also take into account all of the factors affecting gene flow, genetic drift, and hence, the genetic diversity of host populations.

## 2. Impact of Infectious Diseases on Host Populations

Throughout generations, interactions with pathogens produce evolutionary changes in host populations [39,72]. In a host population, pathogen infections favor genotypes with higher resistance (ability to limit pathogen burden [73,74]) or tolerance (ability to limit disease severity induced by a given pathogen burden [73]). Despite the fact that resistance diminishes pathogen virulence and prevalence (see above), tolerance has a nearly neutral effect on pathogen fitness and does not tend to reduce disease spread [75]. In addition to changes in resistance or tolerance, diseases can have other impacts on host populations: population reduction, changes of age structure, alteration on life-history parameters, or effects on genetic diversity [76]. However, the presence of pathogens can also cause changes to host behavior. 

Mate choice is a behavioral process highly influenced by the action of pathogens. For instance, individuals can avoid infected mates to reduce pathogen transmission [77,78,79]. However, host–pathogen interactions have induced evolutionary processes that are responsible for the functioning of other mate choice-related behaviors. Firstly, evolutionary models explain that females choose to mate with males with extravagant ornaments, because these males prove their resistance or tolerance to pathogens [80]. On the other hand, individuals (mainly females [81,82]) can choose genetically dissimilar mates to promote genetic diversity of descendants and, hence, their capacity to resist or tolerate pathogens [83,84,85]. However, the existence of infectious diseases might boost individuals to choose mates with the same level of infection, a behavior that tends to favor genetically similar mating and loss of population genetic diversity [86,87]. 

Studies have also investigated the effects of infectious diseases on dispersal behavior that, in turn, influence the genetic structure of populations [88,89]. Demographic declines that follow disease outbreaks increase resource availability and decrease dispersal advantages. Consequently, the low need for dispersal reduces gene flow and enhances genetic differentiation. However, the expected reduction in genetic diversity as a consequence of low dispersal might be counteracted by the effect of balancing selection acting on immune genes during the disease outbreak [90]. 

Pathogen–host coevolutionary dynamics may be characterized by fluctuating selection (FS), where host genotypes may be at any moment more resistant to contemporary, compared to past or future pathogens, or by arms races (AR), where both hosts and pathogens tend to increase resistance/infectivity over time [91]. The FS dynamic is based on specialized interactions and, hence, it is typical of spatially structured environments. Mixing locally adapted phenotypes may shift the coevolutionary interactions from FS to AR, due to exposure to a higher range of genotypes selected for a wider range of resistance/infectivity in both coevolutionary counterparts [92].

These coevolutionary dynamics can obviously be affected by human management of populations and environments (see below), but also might have effects on the interaction between dispersal and mating behavior. Since spatial variation causes the evolution of locally adaptive immunity, individuals might tend to reduce the contact or refuse mating with genetically different conspecifics harboring dangerous pathogens [93,94]. Genetically different individuals might tolerate pathogens for which local immune systems might not be prepared. They also include genes related to immune system that have not been selected under the local pathogen–host coevolutionary dynamics. These characteristics might make local individuals reluctant to contact and mate with genetically different individuals proceeding from distant populations.

## 3. Ungulates as Hosts

Many human infectious diseases originate from mammals [95,96]. Among mammals, ungulates (a paraphyletic group that includes Artiodactyla and Perissodactyla orders) include a high proportion of wild species with zoonotic diseases. For instance, Han et al. [97] found that 32% of wild ungulate species (73/247 species) were zoonotic hosts. The high rates of disease transmission from wild ungulates to human have been mainly driven by our contact with these species throughout human history [96,98,99]. Ungulates comprise most domestic mammal species. Wild and domestic ungulates can present high levels of contact and relatedness. This contact generates a wildlife–livestock interface where disease transmission has been reported as a precursor to disease emergence in humans [96,100,101,102]. In addition to promoting species conservation, the maintenance of high levels of genetic diversity in wild ungulates should reduce risks regarding the emergence of infectious diseases, these risks being some of the most important threats to human health and the global economy [103,104,105].

Genetic diversity of ungulates is being altered by human-mediated processes acting on gene flow or effective size of populations. Due to hunting, competition with livestock, and lost habitat, many ungulates occur in small or bottleneck populations [106,107]. Anthropogenic barriers, such as highways, block gene flows [108,109,110]. Sex-biased harvesting changes population structures and reduces effective population sizes [111,112]. These processes tend to decrease ungulate genetic diversity and, hence, affect species conservation and the probability of infectious disease emergence and spread. Therefore, ungulate management has relevant implications in conservation and public health prospects. 

**Figure 1:**
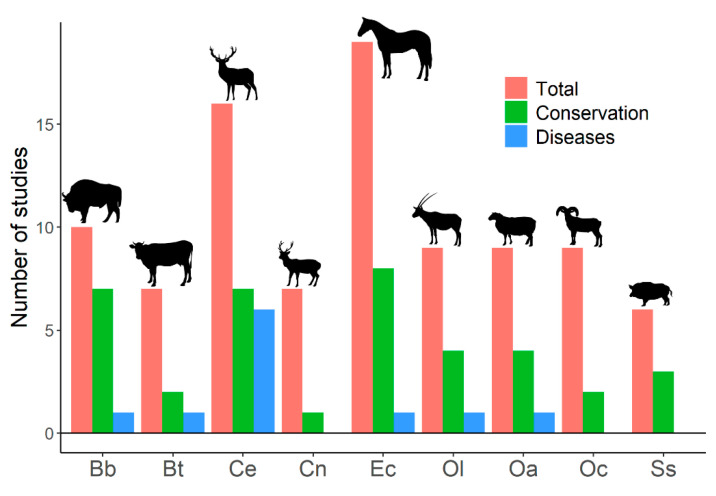
Number of published studies on genetic diversity for the most frequently studied ungulates. Results from a search on the Web of Science with the following search terms: *genetic diversity, inbreeding,* and *ungulates* (217 studies were obtained). Studies on genetic diversity of ungulate populations published in scientific journals were selected (204 papers). Total: number of studies on genetic diversity of ungulate populations published in scientific journals. Conservation: number of studies that explicitly related genetic diversity to conservation (papers in which the word ‘conservation’ appeared in the title, abstract, or the name of the journal). Diseases: number of studies that explicitly associated genetic diversity with diseases (papers in which the title, abstract, or name of the journal used at least one of the following terms: ‘disease’, ‘pathogen’, ‘parasite’, any variation of ‘immunity’, or the name of any disease). Bb: *Bison bonasus*, Bt: *Bos taurus*, Ce: *Cervus elaphus*, Cn: *Cervus nippon*, Ec: *Equus caballus*, Ol: *Oryx leucoryx*, Oa: *Ovis aries*, Oc: *Ovis canadensis*, Ss: *Sus scrofa*. The search was last consulted on 15 April 2021. See Tables S1 and S2.

Despite human-mediated alterations of gene flows and effective population sizes, studies addressing genetic diversity of ungulate populations focus primarily on conservation prospects, rather than on its effects on disease emergence and spread (Figure 1, and Tables S1 and S2). The search on the Web of Science (described in Figure 1) focused on genetic diversity of ungulates and retrieved 204 papers. Only 23 (12.3%) of these papers explicitly associated genetic diversity with diseases. This search showed studies that relate host genetic diversity to the spread of infectious diseases [113] (see below); studies on population genetic structure that highlight the importance of genetic diversity on disease emergence, spread, or development [114,115,116,117,118,119,120,121,122,123]; studies that analyze genetic loci or genetic metrics related to the ability of individuals and population to deal with pathogens and diseases [124,125,126]; studies that associate low genetic diversity with the presence of non-infectious diseases, alterations, or distinctive traits [127,128,129,130,131,132]; and studies on heterozygosity–fitness correlations that show expected [133] or unexpected results [134,135]. Contrarily, out of the 204 found papers, 101 studies (49.5%) explicitly relate genetic diversity to conservation. Therefore, a notable disconnection appears between studies treating ungulate genetic diversity and those dealing with infectious diseases in ungulates. The emergence and spread of infectious diseases, as well as their threats for human health and economy, might be explicitly added to conservation arguments when dealing with genetic diversity of wildlife. This might also help to increase the incorporation of genetic diversity on wildlife management policies that may currently be absent of insufficient [136,137,138].

## 4. Wild Boar, Red Deer, and Tuberculosis

Animal tuberculosis (TB) is a zoonotic infectious disease that affects domestic ungulates and a wide range of wild animals, but it can also be transmitted to humans [139,140]. Because of its effects on wildlife, livestock, and humans, TB represents an important threat to biodiversity, countries’ economies, and public health [141,142]. The causative agents of TB in humans and ungulates are a group of closely related acid-fast bacilli, collectively known as the *Mycobacterium tuberculosis* complex (MTBC [143,144]). *Mycobacterium bovis* and *M. caprae*, both MTBC members are mainly found in domesticated cattle and goats, but they are also frequently isolated from several wild animal species which can act as reservoirs [143]. Additionally, *M. bovis* is the most successful zoonotic pathogen from the MTBC [145,146,147] and it is one of the top 10 causes of death worldwide [148]. 

The relevance of the triad wild boar, red deer, and tuberculosis can be illustrated by the situation in Spanish populations. In Spain, TB caused by *M. bovis* and *M. caprae* have been detected in humans [149,150], and their prevalence in livestock remains high [151]. This high prevalence might be a result of the presence of wild reservoirs of *Mycobacterium* bacilli, mainly wild boar and red deer [152,153,154,155,156,157]. The ecology and behavior of wild reservoirs influence the prevalence and dynamic of the infectious disease [153,158,159]. However, population genetic diversity of these reservoirs might be important for TB prevalence. 

**Figure 2:**
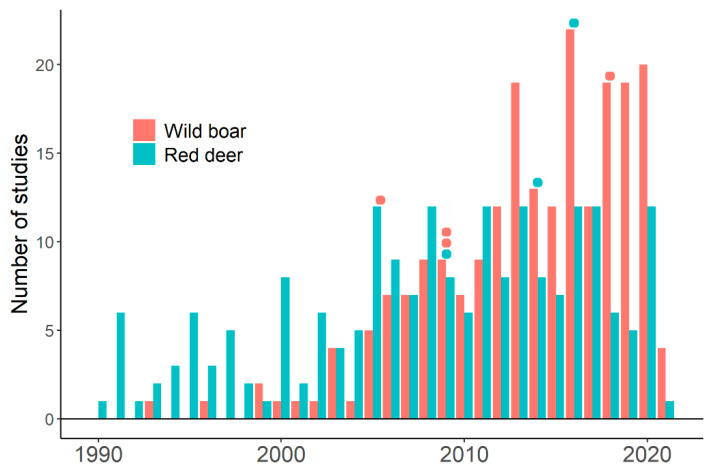
Studies on tuberculosis in wild boar and red deer populations from 1990 to April 2021. Results from the search on the Web of Science described in Table 1 (Selected papers). Colored points indicate the year in which studies explicitly relating tuberculosis to reservoir genetic diversity were published. Red points: studies for wild boar. Blue points: studies for red deer. See Tables S3 and S4.

Genetic diversity of wild boar and red deer populations influences both susceptibilities to TB infection and risk of disease progression. Genetic diversity confers significant resistance to *M. bovis* infection and modulates TB progression in wild boar [160,161]. Genetic diversity of red deer also positively correlates with the ability to control disease progression, inbred populations presenting a higher risk for developing severe TB [113]. These studies highlight the importance of host genetic diversity in the epidemiology of infection. However, studies treating TB in wild boar and red deer rarely consider the role of host genetic diversity on disease emergence or spread (Table 1, Figure 2, and Tables S3 and S4). Table 1 shows that the number of studies that explicitly consider the role of host genetic diversity on disease emergence or spread is four studies for wild boar [160,161,162,163] and three studies for red deer [113,117,120]. These studies relate host genetic diversity to the spread of the infectious disease [113,160,161], analyze population genetic structure and highlight its importance on disease emergence or spread [117,120], and propose methods to detect heterozygosity–fitness associations [162,163]. Scientific bibliography presents little information about the influence of host genetic diversity on a threat to wildlife, livestock, and humans [141,142]. Authorities and wildlife managers might perceive that host genetic diversity might not be a relevant issue to deal with TB in wild boar and red deer populations. Therefore, we could be fighting TB without using all available weapons. Research lines and management guidelines may increase the explicit use of the relationship between reservoirs’ genetic diversity and TB prevalence and spread.

Despite the existing disconnection between studies on genetic diversity and those on the emergence and spread of diseases, the knowledge gathered in each discipline can be applied to the other. Current knowledge regarding factors affecting genetic diversity can be used by managers to fight the spread of disease. Factors that tend to reduce genetic diversity of wildlife present challenges for controlling the prevalence and transmission of infectious diseases. On the other hand, the evolutionary context of disease emergence and transmission might help to understand processes related to genetic diversity. Hereinafter, we will illustrate bidirectional applications in wild boar and red deer populations in Spain, where TB is an important threat for wildlife, livestock, and humans. These applications might be extrapolated to other populations, mainly to those with similar environmental and management conditions. 

## 5. Factors Affecting Wild Boar and Red Deer Genetic Diversity. Recommendations to Confront TB

Wild boar and red deer are important game species in Spain, where most populations are in private hunting estates (typically 750–3000 ha). In these private estates, wild boar and red deer can coexist with other wild ungulates, such as fallow deer, or domestic ungulates, such as cattle [155,164]. Different management actions are conducted to increase hunting harvesting and trophy quality. These management actions alter ecology and behavior of individuals that, in turn, affect gene flow and effective population size. Consequently, some populations can present low levels of genetic diversity or high inbreeding, reference [113,117,160,161,165,166,167,168,169] which are associated with low antler development in red deer [130] and high predisposition to TB progression in both species [113,160,161]. 

A management action, presumably affecting genetic diversity, is the placement of high perimetral fences around the estates to maintain wild ungulates inside the owned land [159,170,171]. As a result of this practice, in some of the properties, we can find two types of hunting estates: open (without perimetral fences) and fenced (with perimetral fences). Hunting and population management in open estates influence the populations occurring in neighboring estates, while in fenced ones, hunting and management are more independent from the activity of neighboring estates. Regardless of management differences, fenced estates are expected to block gene flow [169]. Additionally, small effective population sizes at the moment of fence placement might cause a founder effect with important consequences on genetic diversity and inbreeding. The lack of gene flow and founder effects might make wild boar and red deer in fenced estates present low levels of genetic diversity and might make potentially dangerous populations. If these populations coexist with cattle or goats, the risk of TB transmission and prevalence might be particularly high. In addition to periodically assessments of TB prevalence, periodic controls of genetic diversity of both reservoirs can be recommended in these estates. Despite this general recommendation, there are some considerations that might be taken into account regarding fenced estates. 

To our knowledge, there is no study showing lower levels of genetic diversity of wild boar populations located in fenced and open estates. Additionally, studies comparing red deer populations in open and fenced estates have found that there are not differences in genetic diversity between both types of estates [165,168,169]. Fences might allow a certain degree of individual movements among estates and, hence, genetic diversity in fenced estates can be maintained. Gene flow among fenced estates might be intense for wild boars which have a strong ability to surpass fences. On the contrary, fences have been demonstrated to significantly affect red deer movements [172]. In addition to fence permeability, behavioral processes might avoid the loss of genetic diversity in both species [173,174,175] (see below). In spite of the existence of these processes tending to maintain reservoir genetic diversity, fenced estates, mainly those in which wild reservoirs and livestock coexist, bear potential risks that should be monitored. 

Translocations are relatively common actions in ungulates’ management, mainly to reinforce (or ‘improve’) existing populations or (re)introduce new populations [176,177]. Owners and managers might increase genetic diversity and reduce inbreeding in fenced estates by translocating individuals from different populations [178]. However, translocations imply the existence of additional risks that might overcome their benefits in maintaining reservoir genetic diversity. Hybridization after translocations might cause outbreeding depression due to genetic incompatibility and reduced local adaptation [179,180]. Moreover, translocated individuals swamp the genetic variation of the native populations and, hence, increase homogenization and reduce genetic diversity of a species scale [179,180,181]. Additionally, migrants might put native populations at risk by introducing new pathogens or pathogen strains and altering host–pathogen relationships [182,183,184]. In order to minimize the disadvantages of translocations, owners and managers should select individuals from populations that occur in nearby, similar habitats, and with low genetic divergence [185,186].

In open estates, migration tends to avoid loss of genetic diversity. Wild boar and red deer can move along large distances. Dispersal distances bigger than 50 km have been found for both species [187,188]. However, red deer populations in open and fenced estates of Spain present particularities. In open estates, managers promote the harvesting of the maximum number of males before they are hunted by neighbors. This hunting regime results in populations with mostly young males and strongly female-biased sex ratios [170]. The low proportion of adult males tends to reduce mate competition [171,189] and might tend to reduce migration rates in the typical male-biased dispersal of red deer populations [60,190,191]. The low rate of migration rates of males among open estates might hinder the maintenance of genetic diversity and explain why open and fenced estates do not present different levels of genetic variation. However, in these estates, probably to avoid inbreeding, dispersal has become female-biased [171]. Therefore, genetic diversity in open estates can be, to some extent, maintained by female dispersal. Nevertheless, a reduction in hunting harvesting over males can be recommended to equilibrate population structure and recover natural dispersal of males. 

In addition to fences and population structure, landscape might also influence dispersal and gene flow in wild boar and red deer. Wild boars have high abilities to surpass barriers [108] and their movements might be mainly determined by resource distribution. For red deer, however, landscape features significantly affect movements [192]. In Spain, forest continuity has been shown to favor red deer movements and dispersal [168]. Therefore, to avoid loss of generic diversity in red deer populations, refuge (forest) continuity might be recommended to facilitate individual movements between open estates. 

In addition to processes related to dispersal, game management affects the mating system of Spanish red deer and wild boar populations. Altered red deer population structures in open estates with mostly young males and female-biased sex ratios cause a decrease in male mate competition during the rut [171]. On the contrary, hunting regimes and management in fenced estates maintain equilibrated population structures with the presence of males of all age classes and sex ratios near to 1:1 [170]. Equilibrated sex ratios tend to reduce the effect of genetic drift and to maintain genetic diversity. However, high levels of mate competition in fenced estates favor the success of those males with higher levels of genetic diversity [175]. This selective pressure favors the increase in the genetic diversity contributed by males to the following generation. It is worth highlighting that the genetic diversity contributed by males tends to be higher than that transmitted by females in populations with high levels of mate competition [175]. The higher effective population size and genetic diversity contributed by males might help to explain why red deer populations in fenced estates have similar genetic diversity than that in open estates.

Another mating system related behavior that can be affected by game management is dissimilar mating. Red deer females tend to mate with genetically dissimilar males, predominantly when they produce daughters [174]. This result, which is contextualized under the sexually antagonistic selection [193], also tends to favor the maintaining of genetic diversity. In open estates, the low proportion of males, that are mainly young and philopatric, hinders the action of dissimilar mating. Therefore, dissimilar mating in fenced estates, where the proportion of adult males is high, might favor genetic diversity conservation and might also help to explain the lack of differences in genetic diversity between fenced and open estates. However, this argument loses importance in those open estates where females disperse to avoid inbreeding [171].

Inbreeding depression is an important selective pressure affecting behavioral processes related to mating system [194,195]. These processes tend to compensate the loss of genetic diversity of populations and their action depends on the existence of equilibrated population structures. Male-biased hunting in red deer causes altered population structures that hinder the action of behaviors favoring genetic diversity conservation. Whenever possible, hunting regimes favoring equilibrated population structures in red deer populations might be recommended. 

In wild boar, mating system related processes affecting genetic diversity have been found. Firstly, multiple paternity (different males siring offspring within the same litter) tends to maintain genetic diversity [196,197] and it has been found in wild boar populations [173]. On the other hand, genetic diversity contributed by males to the following generation tends to be higher than that contributed by females [173]. With regard to red deer [175], this result can be due to the advantage of those males with higher levels of genetic diversity during mate competition. Multiple paternity and male mate competition, both tending to favor genetic diversity conservation, are expected to act mainly in equilibrated population structures with high proportion of adult males. Commercial hunting on the wild boar is not so likely as in the red deer to produce biases in population structures. Nevertheless, management actions ensuring equilibrated population structures might be recommended to favor genetic diversity conservation. 

Finally, dissimilar mating has been also assessed for wild boar, but it has not been found. On the contrary, offspring genetic diversity tends to be lower than that expected under random mating [198]. This result has been interpreted as a case of outbreeding avoidance that tends to decrease population genetic diversity throughout generations [198]. In populations where inbred individuals with low genetic diversity have lower resistance to diseases such as TB [160,161], outbreeding avoidance might not make sense. However, in the context of coevolution with pathogens, avoiding genetically dissimilar mates might be beneficial under a scenario of local adaptation and FS dynamics [92]. In addition, outbreeding depression costs might be boosted in environments in which wild boar contact and interbred with domestic pigs in extensive farms and after the release of captive animals [199,200]. Therefore, management actions reducing the contact between wild boar and domestic pigs might be recommended to reduce the selective pressures boosting outbreeding avoidance and loss of genetic diversity in wild boar. 

Studies on genetic diversity of wild boar and red deer populations may yield conclusions applicable by wildlife managers to confront TB. Some recommendations might be summarized as follows:

- Isolated wild boar and red deer populations are potentially dangerous populations.
- When translocations are unavoidable, managers should select individuals from populations that occur nearby, in similar habitats, and with low genetic divergence.
- Mainly in red deer, the continuity of vegetation refuges should be maintained to facilitate individual movements between distant areas.
- Mainly in red deer, sex ratios and male age structures should be equilibrated to favor the natural dispersal of males and the action of evolutionary processes related to the mating system and effective population size.
- In wild boar, the decrease in contact between wild boar and domestic pigs might reduce the selective pressures boosting outbreeding avoidance.

## 6. Disease Transmission and Behavioral Differences between Wild Boar and Red Deer

**Figure 3:**
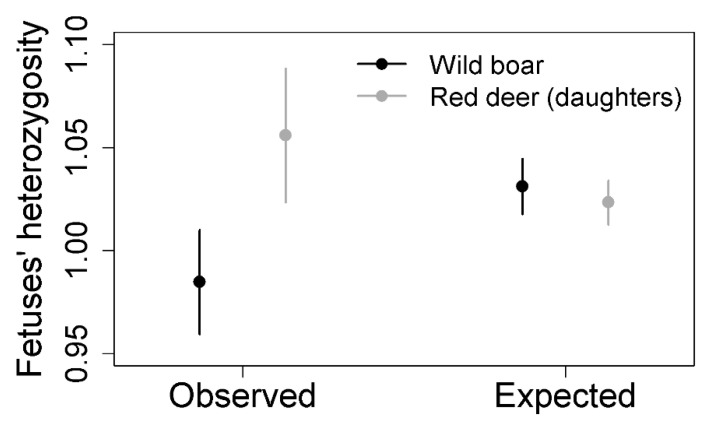
Mean and 95% confidence intervals for observed and expected heterozygosity of fetuses in wild boar and red deer females that produced daughters. Expected heterozygosity was obtained after simulating random mating for each species. See description of the analysis in Table 2.

In central and southwestern Spain, red deer and wild boar coexist in the same habitat and share resources. TB affects both species in which high prevalence and mortality rates have been detected [154,155,159,201,202,203,204,205]. Genetic diversity favors TB resistance and decreases disease progression in both cases [113,160,161]. However, mating preferences regarding genetic dissimilarity is contrary in both species [174,198]. Carranza et al. [174] and Pérez-González et al. [198] used different statistical approaches to study genetic dissimilarity in red deer and wild boar. However, by applying the same statistical method, contrary results in both species are confirmed (Table 2, Figure 3).

When inbred individuals or those with lower levels of genetic diversity have lower fitness, the dissimilar mating of red deer is expected [4,195], but the outbreeding avoidance in wild boar might be unexpected. In addition to the evolutionary context of wild boar in which hybridization events with domestic pigs have occurred [198], the probability of disease transmission might help to explain the unexpected results regarding outbreeding avoidance. Therefore, the different behaviors of wild boar and red deer regarding genetically dissimilar mating might be explained after considering the impact of diseases on the evolutionary context of both species. 

Low precipitation and high temperatures during summer have important consequences on resource availability of the Spanish Mediterranean ecosystem [206,207]. During this season, water points are scarce and become places in which fauna contact and aggregate. Wild boar activity around water points is intense [159,208]. Wild boars use water points to drink or wallow. The areas around water points are used for foraging, brushing, or mating. These activities can be accompanied by actions such as urinating or defecating. Consequently, infectious diseases such as TB may be strongly spread from interactions at these water points. TB spread might occur by ingestion or inhalation of nasal and oral excretions from infected individual [158,209]. 

Red deer also uses water points to drink, wallow, or brush. Mating behavior in red deer may also take place near these locations [210,211], which might favor the moisture for vegetation growth during the dry conditions in which rutting season occurs in Mediterranean ecosystems. As in wild boar, water points favor TB spread in red deer populations [159]. However, a particularity has been found regarding the relationship between water points and TB spreading in both species. Vicente et al. [159] found that the risk of TB infection in both wild boar and red deer mainly depends on wild boar aggregation in water points. Wild boar activities and behaviors around water points might cause higher rates of pathogen excretion, and those excreted pathogens might cause TB infection in wild boar, red deer, cattle, and other species. 

High rates of pathogen excretion and the sharing of behaviors, natural cycles, and ecological niche of all individuals of the same species can make wild boar present high levels of contact with pathogens. Accordingly, the prevalence of TB in wild boar populations tends to be higher than that in other ungulates, including red deer [140,154,155,164,204,212]. Wild boar populations present a widespread exposure to TB which can cause high mortality rates [201]. 

The high exposure of wild boar populations to TB may have evolutionary consequences. For instance, wild boar is expected to present high levels of disease resistance and tolerance. Accordingly, studies show higher levels of TB resistance in wild boar than in red deer [154]; infected wild boar showing less serious damages than infected red deer [213]. However, this high exposure of wild boar to TB might impact its behavior by causing a trade-off regarding the dissimilar mating. 

Due to the existence of inbreeding depression [160,161], genetic dissimilarity in mating preferences might be expected in wild boar populations [83,84,85]. However, the existence of other selective pressures might boost individuals to avoid genetically dissimilar mates. For instance, assortative mating regarding the level of infection might cause genetically similar mating and loss of population genetic diversity [86,87]. Moreover, local individuals might avoid the mating with genetically different individuals harboring new pathogens [93,94]. 

In wild boar, the outcome of the trade-off regarding mating with genetically dissimilar or similar individuals might depend on the prevalence of infectious diseases, such as TB [87], and this outcome has a high importance on genetic diversity conservation of populations [86]. This trade-off deserves further research.

## Figures and Tables

**Table 1:** Studies on tuberculosis and host genetic diversity of wild boar and red deer. Results from searches on the Web of Science for both species. For wild boar, the following search terms were used: *wild boar*, *Sus scrofa,* and *tuberculosis*. For red deer, the following search terms were used: *red deer, Cervus elaphus,* and *tuberculosis*. Total: number of obtained studies. Selected: number of studies on tuberculosis published in scientific journals. Journals: number of journals in which the selected papers were published. Genetic diversity: number of selected studies that explicitly relate tuberculosis to reservoir genetic diversity (papers in which the title, abstract, or name of the journal used at least one of the following terms in relation to host populations: ‘genetic diversity’, ‘genetic variability’, ‘genetic variation’, ‘genomic diversity’, ‘genomic variability’, ‘genomic variation’, ‘inbreeding’, ‘heterozygosity’, ‘heterozygosity–fitness’, or ‘heterosis’). Percentage: percentage of studies that explicitly relate tuberculosis to reservoir genetic diversity in relation to the selected studies. Searches were last consulted on 15 April 2021. See Figure 2, and Tables S3 and S4.

Species	Total	Selected	Journals	Genetic Diversity	Percentage
Wild boar	299	217	74	4	1.8
Red deer	282	215	69	3	1.4

**Table 2:** Mating preferences regarding genetic dissimilarity in wild boar and red deer from Iberian Peninsula. Analysis conducted with data from Pérez-González et al. [198] and Carranza et al. [174]. Both studies conducted different approaches to assess dissimilar mating. Here, both datasets were analyzed with the same procedure (see [198]). For red deer, data from females producing daughters were selected, because dissimilar mating was only obtained for this type of female (see [174]). In order to determine the existence of dissimilar mating, we assessed the genetic relationship between parents using the standardized heterozygosity [32] of the fetuses. We considered that dissimilar mating occurred when the observed heterozygosity of fetuses was higher than expected under random mating. To simulate random mating, we randomly combined the genotypes of females (mothers) and males from the same hunting event. We randomly selected a haploid genotype of a female and a haploid genotype of a male from the same hunting event to create a diploid simulated offspring. This process was repeated 1000 times for each female and the heterozygosity of its simulated offspring was quantified. The mean heterozygosity of the 1000 simulated offspring was considered as the expected heterozygosity under random mating for this female. Therefore, each female had two variables: observed heterozygosity of its offspring (1 value for red deer, and as many values as fetuses it gestated for wild boar) and expected heterozygosity under random mating (1 value for both species). Observed and expected heterozygosity were compared using a linear mixed-effect model (LME) fitted by reduced maximum likelihood, with heterozygosity as dependent variable, mating type (observed vs. expected) and species (red deer and wild boar) and the interaction of both as fixed factors, and female within hunting event as nested random effects. Table shows the LME results for the comparison between observed and expected heterozygosity in fetuses for wild boar and red deer. Wild boar and expected heterozygosity as references. See Figure 3.

	Value	SE	DF	t-Value	*p*-Value
Intercept	1.033	0.021	300	49.648	<0.001
Mating type	−0.047	0.022	250	−2.133	0.034
Species	−0.010	0.026	21	−0.376	0.710
Mating type × Species	0.079	0.030	250	2.639	0.009

## Data Availability

The data presented in this study are available on request from the corresponding author.

## Supplementary Materials

The following are available online at https://www.mdpi.com/article/10.3390/ani11061630/s1, Table S1: Number of studies on genetic diversity for ungulate species, Table S2: Number of studies in scientific journals (ungulates), Table S3: Number of studies in scientific journals (wild boar), Table S4: Number of studies in scientific journals (red deer).

## References

[1] Kohn M.H., Murphy W.J., Ostrander E.A., Wayne R. (2006). Genomics and conservation genetics. Trends Ecol. Evol..

[2] Frankel O.H. (1974). Genetic conservation: Our evolutionary responsibility. Genetics.

[3] Frankel O.H. (1970). Variation, the essence of life. Proc. Linn. Soc. N. S. W..

[4] Charlesworth B., Charlesworth D. (1999). The genetic basis of inbreeding depression. Genet. Res..

[5] Frankel O.H., Soulé M.E. (1981). Conservation and Evolution.

[6] Neaves L.E., Eales J., Whitlock R., Hollingsworth P.M., Buerke T., Pullin A.S. (2015). The fitness consequences of inbreeding in natural populations and their implications for species conservation—A systematic map. Environ. Evid..

[7] Frankham R. (2008). Genetic adaptation to captivity in species conservation programs. Mol. Ecol..

[8] Frankham R. (2005). Genetics and extinction. Biol. Conserv..

[9] Amos W., Balmford A. (2001). When does conservation genetics matter?. Heredity.

[10] O’Brien S.J. (1994). A role for molecular genetics in biological conservation. Proc. Natl. Scad. Sci. USA.

[11] Morse S.S., Mazet J.A., Woolhouse M., Parrish C.R., Carroll D., Karesh W.B., Zambrana-Torrelio C., Lipkin W.I., Daszak P. (2012). Prediction and prevention of the next pandemic zoonosis. Lancet.

[12] Jones K.E., Patel N.G., Levy M.A., Storeygard A., Balk D., Gittleman L., Daszak P. (2008). Global trends in emerging infectious diseases. Nature.

[13] Kruse H., Kirkemo A.-M., Handeland K. (2004). Wildlife as source of zoonotic Infections. Emerg. Infect. Dis..

[14] Taylor L.H., Latham S.M., Woolhouse M.E.J. (2001). Risk factors for human disease emergence. Philos. Trans. R. Soc. Lond. B Biol. Sci..

[15] Allen T., Murray K.A., Zambrana-Torrelio C., Morse S.S., Rondinini C., Di Marco M., Breit N., Olival K.J., Daszak P. (2017). Global hotspots and correlates of emerging zoonotic diseases. Nat. Commun..

[16] Heymann D.L., Chen L., Takemi K., Fidler D.P., Tappero J.W., Thomas M.J., Kenyon T.A., Frieden T., Yach D., Nishtar S. (2015). Global health security: The wider lessons from the west African Ebola virus disease epidemic. Lancet.

[17] Morens D.M., Fauci A.S. (2012). Emerging infectious diseases in 2012: 20 years after the institute of medicine report. Mbio.

[18] Lu H., Stratton C.W., Tang Y. (2020). Outbreak of pneumonia of unknown etiology in Wuhan China: The mystery and the miracle. J. Med. Virol..

[19] McKibbin W., Fernando R. (2021). The Global Macroeconomic Impacts of COVID-19: Seven Scenarios. Asian Econ. Pap..

[20] Sohrabi C., Alsafi Z., O’Neill N., Khan M., Kerwan A., Al-Jabir A., Iosifidis C., Agha R. (2020). World Health Organization declares global emergency: A review of the 2019 novel coronavirus (COVID-19). Int. J. Surg..

[21] Pike J., Bogich T.L., Elwood S., Finnoff D.C., Daszak P. (2014). Economic optimization of a global strategy to reduce the pandemic threat. Proc. Natl. Acad. Sci. USA.

[22] Quéméré E., Rossi S., Petit E., Marchard P., Merlet J., Game Y., Galan M., Gilot-Fromont E. (2020). Genetic epidemiology of the Alpine ibex reservoir of persistent and virulent brucellosis outbreak. Sci. Rep..

[23] Portanier E., Garel M., Devillard S., Maillard D., Poissant J., Galan M., Benabed S., Poirel M.T., Duhayer J., Itty C. (2019). Both candidate gene and neutral genetic diversity correlate with parasite resistance in female Mediterranean mouflon. BMC Ecol..

[24] Mitchell J., Vitikainen E.I.K., Wells D.A., Cant M.A., Nichols H.J. (2017). Heterozygosity but not inbreeding coefficient predicts parasite burdens in the banded mongoose. J. Zool..

[25] Benavides M.V., Sonstegard T.S., Van Tassell C. (2016). Genomic regions associated with sheep resistance to gastrointestinal nematodes. Trends Parasitol..

[26] Sweeney T., Hanrahan J.P., Ryan M.T., Good B. (2016). Immunogenomics of gastrointestinal nematode infection in ruminants—Breeding for resistance to produce food sustainably and safely. Parasite Immunol..

[27] Hayward A.D. (2013). Causes and consequences of intra- and inter-host heterogeneity in defense against nematodes. Parasite Immunol..

[28] Ruiz-López M.J., Monello R.J., Gompper M.E., Eggert L.S. (2012). The effect and relative importance of neutral genetic diversity for predicting parasitism varies across parasite taxa. PLoS ONE.

[29] Saddiqi H.A., Jabbar A., Sarwar M., Iqbal Z., Muhammad G., Nisa M., Shahzad A. (2011). Small ruminant resistance against gastrointestinal nematodes: A case of *Haemonchus contortus*. Parasitol. Res..

[30] Acevedo-Whitehouse K., Gulland F., Greig D., Amos W. (2003). Disease susceptibility in California sea lions. Nature.

[31] Cassinello J., Gomendio M., Roldan E.R.S. (2001). Relationship between coefficient of inbreeding and parasite burden in endangered gazelles. Conserv. Biol..

[32] Coltman D.W., Pilkington J.G., Smith J.A., Pemberton J.M. (1999). Parasite-mediated selection against inbred Soay sheep in a free-living, island population. Evolution.

[33] Hansson B., Westerberg L. (2002). On the correlation between heterozygosity and fitness in natural populations. Mol. Ecol..

[34] Bateson Z.W., Hammerly S.C., Johnson J.A., Morrow M.E., Whittingham L.A., Dunn P.O. (2016). Specific alleles at immune genes, rather than genome-wide heterozygosity, are related to immunity and survival in the critically endangered Attwater’s prairie chicken. Mol. Ecol..

[35] Brambilla A., Biebach I., Bassano B., Bogliani G., von Hardenberg A. (2015). Direct and indirect causal effects of heterozygosity on fitness-related traits in Alpine ibex. Proc. R. Soc. B Biol. Sci..

[36] Janeway C.A., Travers P., Walport M., Shlomchik M.J., Janeway C. (2001). The major histocompatibility complex and its functions. The Immune System in Health and Disease.

[37] Aguilar A., Roemer G., Debenham S., Binns M., Garcelon D., Waine R.K. (2004). High MHC diversity maintained by balancing selection in an otherwise genetically monomorphic mammal. Proc. Natl. Scad. Sci. USA.

[38] Hughes A.L., Hughes M.K., Howell C.Y., Nei M. (1994). Natural selection at the class II major histocompatibility complex loci of mammals. Philos. Trans. R. Soc. Lond. B Biol. Sci..

[39] Hedrick P.W., Thomson G. (1983). Evidence for balancing selection at HLA. Genetics.

[40] Spurgin L.G., Richardson D.S. (2010). How pathogens drive genetic diversity: MHC, mechanisms and misunderstandings. Proc. R. Soc. B Biol. Sci..

[41] Acevedo-Whitehouse K., Cunningham A.A. (2006). Is MHC enough for understanding wildlife immunogenetics?. Trends Ecol. Evol..

[42] Bernatchez L., Landry C. (2003). MHC studies in nonmodel vertebrates: What have we learned about natural selection in 15 years?. J. Evol. Biol..

[43] Turner A.K., Begon M., Jackson J.A., Paterson S. (2012). Evidence for selection at cytokine loci in a natural population of field voles (*Microtus agrestis*). Mol. Ecol..

[44] Robinson S.J., Samuel M.D., Johnson C.J., Adams M., McKenzie D.I. (2012). Emerging prion disease drives host selection in a wildlife population. Ecol. Appl..

[45] Johnson C., Johnson J., Vanderloo J.P., Keane D., Aiken J.M., McKenzie D. (2006). Prion protein polymorphisms in white-tailed deer influence susceptibility to chronic wasting disease. J. Gen. Virol..

[46] White P.S., Choi A., Pandey R., Menezes A., Penley M., Gibson A.K., de Roode J., Morran L. (2020). Host heterogeneity mitigates virulence evolution. Biol. Lett..

[47] Morley D., Broniewski J.M., Westra E.R., Buckling A., van Houte S. (2017). Host diversity limits the evolution of parasite local adaptation. Mol. Ecol..

[48] Altizer S., Harvell D., Friedle E. (2003). Rapid evolutionary dynamics and disease threats to biodiversity. Trends Ecol. Evol..

[49] Regoes R.R., Nowak M.A., Bonhoeffer S. (2000). Evolution of virulence in a heterogeneous host population. Evolution.

[50] Schmid-Hempel P. (1998). Parasites in Social Insects.

[51] Anderson R.M., May R.M. (1986). The invasion, persistence and spread of infectious diseases within animal and plant communities. Philos. Trans. R. Soc. Lond. B Biol. Sci..

[52] Ekroth A.K.E., Rafaluk-Mohr C., King K.C. (2019). Host genetic diversity limits parasite success beyond agricultural systems: A meta-analysis. Proc. R. Soc. B Biol. Sci..

[53] van Houte S., Ekroth A.K., Broniewski J.M., Chabas H., Ashby B., Bondy-Denomy J., Gandon S., Boots M., Paterson S., Buckling A. (2016). The diversity-generating benefits of a prokaryotic adaptive immune system. Nature.

[54] King K.C., Lively C.M. (2012). Does genetic diversity limit disease spread in natural host populations?. Heredity.

[55] Campbell G., Noble L.R., Rollinson D., Southgate V.R., Webster J.P., Jones C.S. (2010). Low genetic diversity in a snail intermediate host (*Biomphalaria pfeifferi* Krass, 1848) and schistosomiasis transmission in the Senegal River Basin. Mol. Ecol..

[56] Bani L., Orioli V., Pisa G., Dondina O., Fagiani S., Fabbri E., Randi E., Mortelliti A., Sozio G. (2017). Landscape determinants of genetic differentiation, inbreeding and genetic drift in the hazel dormouse (*Muscardinus avellanarius*). Conserv. Genet..

[57] Gubili C., Mariani S., Weckworth B.V., Galpern P., McDevitt A.D., Hebblewhite M., Nickel B., Musiani M. (2017). Environmental and anthropogenic drivers of connectivity patterns: A basis for prioritizing conservation efforts for threatened populations. Evol. Appl..

[58] Charlesworth B. (2009). Effective population size and patterns of molecular evolution and variation. Nat. Rev. Genet..

[59] Epps C.W., Wehausen J.D., Bleich V.C., Torres S.G., Brashares J.S. (2007). Optimizing dispersal and corridor models using landscape genetics. J. Appl. Ecol..

[60] Lawson Handley J.L., Perrin N. (2007). Advances in our understanding of mammalian sex-biased dispersal. Mol. Ecol..

[61] Briton J., Nurthen R.K., Briscoe D.A., Frankham R. (1994). Modelling problems in conservation genetics using *Drosophila*: Consequences of harem. Biol. Conserv..

[62] Slatkin M. (1987). Gene flow and the geographic structure of natural populations. Science.

[63] Nei M. (1973). Analysis of gene diversity in subdivided populations. Proc. Natl. Scad. Sci. USA.

[64] Wright S. (1938). Size of population and breeding structure in relation to evolution. Science.

[65] Arauco-Shapiro G., Schmacher K.I., Boersma D., Bouzat J.L. (2020). The role of demographic history and selection in shaping genetic diversity of the Galápagos penguin (*Spheniscus mendiculus*). PLoS ONE.

[66] Bouzat J.L. (2010). Conservation genetics of population bottlenecks: The role of change, selection, and history. Conserv. Genet..

[67] Hedrick P.W. (2005). Genetic of Populations.

[68] Frankham R., Ballou J.D., Briscoe D.A. (2002). Introduction to Conservation Genetics.

[69] Hedrick P.W., García-Dorado A. (2016). Understanding inbreeding depression, purging, and genetic rescue. Trends Ecol. Evol..

[70] Groombridge J.J., Jones C.G., Bruford M.W., Nichols R.A. (2000). ‘Ghost’ alleles of the Mauritius kestrel. Nature.

[71] Bonnell M.L., Selander R.L. (1974). Elephant seals: Genetic variation and near extinction. Science.

[72] Hamilton W.D., Axelrod R., Tanese R. (1990). Sexual reproduction as an adaptation to resist parasites (a review). Proc. Natl. Acad. Sci. USA.

[73] Råberg L., Sim D., Read A.F. (2007). Disentangling genetic variation for resistance and tolerance to infectious diseases in animals. Science.

[74] Bishop S.C., Stear M.J. (2003). Modeling of host genetics and resistance to infectious diseases: Understanding and controlling nematode infections. Vet. Parasitol..

[75] Roy B., Kirchner J. (2000). Evolutionary dynamics of pathogen resistance and tolerance. Evolution.

[76] Blanchong J.A., Robinson S.J., Samuel M.D., Foster J.T. (2016). Application of genetics and genomics to wildlife epidemiology. J. Wildl. Manage..

[77] Martinez-Padilla J., Vergara P., Mougeot F., Redpath S.M. (2012). Parasitized mates increase infection risk for partners. Am. Nat..

[78] Arakawa H., Cruz S., Deak T. (2011). From models to mechanisms: Odorant communication as a key determinant of social behavior in rodents during illness-associated states. Neurosci. Biobehav. Rev..

[79] Hillgarth N. (1996). Ectoparasite transfer during matins in ring-necked pheasants *Phasianus colchicus*. J. Avian Biol..

[80] Hamilton W.D., Zuk M. (1982). Heritable true fitness and bright birds: A role for parasites?. Science.

[81] Clutton-Brock T.H. (1991). The Evolution of Parental Care.

[82] Trivers R.L., Campbell B. (1972). Parental investment and sexual selection. Sexual Selection and the Descent of Man: 1871–1971.

[83] Setchell J.M., Charpentier M.J.E., Abbot K.M., Wickings E.J., Knapp L.A. (2010). Opposites attract: MHC-associated mate choice in a polygynous primate. J. Evol. Biol..

[84] Penn D.J. (2002). The scent of genetic compatibility: Sexual selection and the major histocompatibility complex. Ethology.

[85] Penn D.J., Potts W.K. (1999). The evolution of mating preferences and major histocompatibility complex genes. Am. Nat..

[86] Campbell L.J., Head M.L., Wilfert L., Griffiths A.G.F. (2017). An ecological role for assortative mating under infection?. Conserv. Genet..

[87] Teacher A.G.F., Garner T.W.J., Nichols R.A. (2009). Population genetic patterns suggest a behavioural change in wild common frogs (*Rana temporaria*) following disease outbreaks (Ranavirus). Mol. Ecol..

[88] Bruniche-Olsen A., Burridge C.P., Austin J.J., Jones M.E. (2013). Disease induced changes in gene flow patterns among Tasmanian devil populations. Biol. Conserv..

[89] Lachish S., McCallum H., Jones M. (2009). Demography, disease and the devil: Life-history changes in a disease-affected population of Tasmanian devils (*Sarcophilus harrisii*). J. Anim. Ecol..

[90] Serieys L.E.K., Lea A., Pollinger J.P., Riley S.P.D., Wayne R.K. (2015). Disease and freeways drive genetic change in urban bobcat populations. Evol. Appl..

[91] Van Valen L. (1973). A new evolutionary law. Evol. Theory.

[92] Gómez P., Ashby B., Buckling A. (2015). Population mixing promotes arms race host-parasite coevolution. Proc. R. Soc. B Biol. Sci..

[93] Thornhill R., Fincher C.L. (2013). The parasite-driven-wedge model of parapatric speciation. J. Zool..

[94] Fincher C.L., Thornhill R. (2008). A parasite-driven wedge: Infectious diseases may explain language and other biodiversity. Oikos.

[95] Woolhouse M.E.J., Gowtage-Sequeria S. (2005). Host range and emerging and reemerging pathogens. Emerg. Infect. Dis..

[96] Cleaveland S., Laurenson M.K., Taylor L.H. (2001). Diseases of humans and their domestic mammals: Pathogen characteristics, host range and the risk of emergence. Philos. Trans. R. Soc. Lond. B Biol. Sci..

[97] Han B.A., Kramer A.M., Drake J.M. (2016). Global patterns of zoonotic disease in mammals. Trends Parasitol..

[98] Lloyd-Smith J.O., George D., Pepin K.M., Pitzer V.E., Pulliam J.R.C., Dobson A.P., Hudson P.J., Grenfell B.T. (2009). Epidemic dynamics at the human-animal interface. Science.

[99] Wolfe N.D., Dunavan C.P., Diamond J. (2007). Origins of major human infectious diseases. Nature.

[100] Wiethoelter A.K., Beltrán-Alcrudo D., Kock R., Mor S.M. (2015). Global trends in infectious diseases at the wildlife–livestock interface. Proc. Natl. Acad. Sci. USA.

[101] Patz J.A., Olson S.H., Uejio C.K., Gibbs H.K. (2008). Disease emergence from global climate and land use change. Med. Clin. North. Am..

[102] Cleaveland S., Haydon D.T., Taylor L. (2007). Overviews of pathogen emergence: Which pathogens emerge, when and why?. Curr. Top. Microbiol. Inmunol..

[103] Dehove A., Commault J., Petitclerc M., Teissier M., Macé J. (2012). Economic analysis and costing of animal health: A literature review of methods and importance. Rev. Sci. Tech..

[104] Williams E.S., Yuill T., Artois M., Fischer J., Haigh S.A. (2002). Emerging infectious diseases in wildlife. Rev. Sci. Tech..

[105] Daszak P., Cunningham A.A.A., Hyatt A.D. (2000). Emerging infectious diseases of wildlife—Threats to biodiversity and Human health. Science.

[106] Ripple W.J. (2015). Collapse of the world’s largest herbivores. Sci. Adv..

[107] Linnell J.D.C., Zachos F.E., Putman R., Apollonio M., Andersen R. (2011). Status and distribution patterns of European ungulates: Genetics, population history and conservation. Ungulate Management in Europe: Problems and Practices.

[108] Frantz A.C., Bertouille S., Eloy M.C., Licoppe A., Chaumont F., Flamand M.C. (2012). Comparative landscape genetic analyses show a Belgian motorway to be a gene flow barrier for red deer (*Cervus elaphus*), but not wild boars (*Sus scrofa*). Mol. Ecol..

[109] Kuehn R., Hindenlang K.E., Holzgang O., Senn J., Stoeckle B., Sperisen C. (2007). Genetic effect of transportation infrastructure on roe deer populations (*Capreolus capreolus*). J. Hered..

[110] Epps C.W., Palsbøll P.J., Wehausen J.D., Roderick G.K., Ramey R.R., McCullough D.R. (2005). Highways block gene flow and cause a rapid decline in genetic diversity of desert bighorn sheep. Ecol. Lett..

[111] Sæther B.-E., Engen S., Solberg E.J. (2009). Effective size of harvested ungulate populations. Anim. Conserv..

[112] Coltman D.W. (2008). Molecular ecological approaches to studying the evolutionary impact of selective harvesting in wildlife. Mol. Ecol..

[113] Queirós J., Vicente J., Alves P.C., de la Fuente J., Gortazar C. (2016). Tuberculosis, genetic diversity and fitness in the red deer, Cervus elaphus. Infect. Genet. Evol..

[114] Kotzé A., Smith R.M., Moodley Y., Luikart G., Birss C., Van Wyk A.M., Dalton D.L. (2019). Lessons for conservation management: Monitoring temporal changes in genetic diversity of cape mountain zebra (*Equus zebra zebra*). PLoS ONE.

[115] Frantz A.C., Zachos F.E., Bertouille S., Eloy M.C., Colyn M., Flamand M.C. (2017). Using genetic tolos to estimate the prevalence of non-native red deer (*Cervus elaphus*) in a western European population. Ecol. Evol..

[116] Strucken E.M., Lee S.H., Jang G.W., Porto-Neto L.R., Gondro C. (2015). Towards breed formation by island model divergence in Korean cattle. BMC Evol. Biol..

[117] Queirós J., Vicente J., Boadella M., Gortázar C., Alves P.C. (2014). The impact of management practices and past demographic history on the genetic diversity of red deer (*Cervus elaphus*): An assessment of population and individual fitness. Biol. J. Linn. Soc..

[118] Smitz N., Corneli D., Chardonnet P., Caron A., de Garine-Wichatitsky M., Jori F., Mouton A., Latinne A., Pigneur L.M., Melletti M. (2014). Genetic structure of fragmented southern populations of African Cape buffalo (*Syncerus caffer caffer*). BMC Evol. Biol..

[119] Wan Q.H., Zhang P., Ni X.W., Wu X.W., Chen Y.Y., Kuang Y.Y., Ge Y.F., Fang S.G. (2011). A novel HURRAH protocol reveals high numbers of monomorphic MHC class II loci and tow asymmetric multi-locus haplotypes in the Pere David’s deer. PLoS ONE.

[120] Fernández-de-Mera I.G., Vicente J., Pérez de la Lastra J.M., Mangold A.J., Naranjo V., Fierro Y., de la Fuente J., Gortázar C. (2009). Reduced major histocompatibility complex class II polymorphism in a hunter-managed isolated Iberian red deer population. J. Zool..

[121] Wojcik J.M., Kawalko A., Tokarska M., Jaarola M., Vallenback P., Pertodi C. (2009). Post-bottleneck mtDNA diversity in a free-living population of European bison: Implications for conservation. J. Zool..

[122] Wilson G.A., Nishi J.S., Elkin B.T., Strobeck C. (2005). Effects of a recent founding event and intrinsic population dynamics on genetic diversity in an ungulate population. Conserv. Genet..

[123] Hedrick P.W., Parker K.M., Gutiérrez-Espeleta G.A., Rattink A., Lievers K. (2000). Major histocompatibility complex variation in the Arabian oryx. Evolution.

[124] Kumar D.R., Devadasan M.J., Surya T., Vineeth M.R., Choudhary A., Sivalingam J., Kataria R.S., Niranjan S.K., Tantia M.S., Verma A. (2020). Genomic diversity and selection sweeps identified in Indian swamp buffaloes reveals it’s uniqueness with riverine buffaloes. Genomics.

[125] McDonald D.B., Hobson E.A. (2018). Edge weight variance: Population genetic metrics for social network analysis. Anim. Behav..

[126] Janova E., Futas J., Klumplerova M., Putnova L., Vrtkva I., Vyskocil M., Frolkova P., Horin P. (2013). Genetic diversity and conservation in a small endangered horse population. J. Appl. Genet..

[127] Larison B., Kaelin C.B., Harrigan R., Henegar C., Rubenstein D.I., Kamath P., Aschenborn O., Smith T.B., Barsh G.S. (2021). Population structure, inbreeding and stripe pattern abnormalities in plains zebras. Mol. Ecol..

[128] Coetzer W.G., Grobler J.P. (2019). Genetic variation among different springbok (*Antidorcas marsupialis*) colour variants. Mol. Ecol..

[129] Sasidharan S.P., Ludwig A., Harper C., Moodley Y., Bertschinger H.J., Guthrie A.J. (2011). Comparative genetics of sarcoid tumour-affected and non-affected mountain zebra (*Equus zebra*) populations. S. Afr. J. Wildl. Res..

[130] Pérez-González J., Carranza J., Torres-Porras J., Fernández-García J.L. (2010). Low heterozygosity at microsatellite markers in Iberian red deer with small antlers. J. Hered..

[131] Marais H.J., Nel P., Bertschinger H.J., Schoeman J.P., Zimmerman D. (2007). Prevalence and body distribution of sarcoids in South African Cape mountain zebra (*Equus zebra zebra*). J. S. Afr. Vet. Assoc..

[132] Zachos F.E., Althoff C., von Steynitz Y., Eckert I., Hartl G.B. (2007). Genetic analysis of an isolated red deer (*Cervus elaphus*) population showing signs of inbreeding depression. Eur. J. Wildl. Res..

[133] Da Silva A., Gaillard J.M., Yoccoz N.G., Hewison A.J.M., Galan M., Coulson T., Allaine D., Vial L., Delorme D., Van Laere G. (2009). Heterozygosity-fitness correlations revealed by neutral and candidate gene markers in roe deer from a long-term study. Evolution.

[134] Kaeuffer R., Reale D., Pontier D., Chapuis J.L., Coltman D.W. (2008). Local effects of inbreeding on embryo number and consequences for genetic diversity in Kerguelen mouflon. Biol. Lett..

[135] Latch E.K., Amann R.P., Jacobson J.P., Rhodes O.E. (2008). Competing hypotheses for the etiology of cryptorchidism in sitka black-tailed deer: An evaluation of evolutionary alternatives. Anim. Conserv..

[136] Laikre L., Hoban S., Bruford M.W., Segelbacher G., Allendorf F.W., Gajardo G., Rodríguez A.G., Hedrick P.W., Heuertz M., Hohenlohe P.A. (2020). Post-2020 goals overlook genetic diversity. Science.

[137] Ralls K., Ballou J.D., Dudash M.R., Eldridge M.D.B., Fenster C.B., Lacy R.C., Sunnucks P., Frankham R. (2018). Call for a Paradigm Shift in the Genetic Management of Fragmented Populations. Conserv. Lett..

[138] Hoban S.M., Hauffe H.C., Pérez-Espona S., Arntzen J.W., Bertorelle G., Bryja J., Frith K., Gaggiotti O.E., Galbusera P., Godoy J.A. (2013). Bringing genetic diversity to the forefront of conservation policy and management. Conserv. Genet. Res..

[139] Pereira A.C., Reis A.C., Ramos B., Cunha M.V. (2020). Animal tuberculosis: Impact of disease heterogeneity in transmission, diagnosis and control. Transbound. Emerg. Dis..

[140] Gortázar C., Torres J., Vicente J., Acevedo P., Reglero M., de la Fuente J., Negro J.J., Aznar-Martin J. (2008). Bovine tuberculosis in Doñana Biosphere Reserve: The role of wild ungulates as disease reservoirs in the last Iberian lynx strongholds. PLoS ONE.

[141] Rodríguez-Campos S., Smith N.H., Boniotti M.B., Aranaz A. (2014). Overview and phylogeny of *Mycobacterium tuberculosis* complex organisms: Implications for diagnostics and legislation of bovine tuberculosis. Res. Vet. Sci..

[142] Harris K.A., Downs S.H., Goodchild A.V., Broughan J.M., Upton P.A., Smith N.H. (2014). Bovine TB infection status in cattle in Great Britain in 2012. Vet. Rec..

[143] Malone K.M., Gordon S.V. (2017). *Mycobacterium tuberculosis* complex members adapted to wild and domestic animals. Adv. Exp. Med. Biol..

[144] Brites D., Gagneux S. (2017). The nature and evolution of genomic diversity in the *Mycobacterium tuberculosis* complex. Adv. Exp. Med. Biol..

[145] Galagan J.E. (2014). Genomic insights into tuberculosis. Nat. Rev. Genet..

[146] Pepperell C.S., Casto A.M., Kitchen A., Granka J.M., Conerejo O.E., Holmes E.C., Birren B., Galagan J., Feldman M.W. (2013). The role of selection in shaping diversity of natural *M. tuberculosis* populations. PLoS Pathog..

[147] Gagneux S. (2012). Host-pathogen coevolution in human tuberculosis. Philos. Trans. R. Soc. B.

[148] (2019). World Tuberculosis Report 2019.

[149] Nebreda T., Álvarez-Prida E., Blanco B., Remacha M.A., Samper S., Jiménez M.S. (2016). Peritoneal tuberculosis due to *Mycobacterium caprae*. IDCases.

[150] Rodríguez E., Sánchez L.P., Pérez S., Herrera L., Jiménez M.S., Samper S., Iglesias M.J. (2009). Human tuberculosis due to *Mycobacterium bovis* and *M. caprae* in Spain, 2004–2007. Int. J. Tuberc. Lung. Dis..

[151] Pérez-Morote R., Pontones-Rosa C., Gortázar-Schmidt C., Muñoz-Cardona A.I. (2020). Quantifying the economic impact of bovine tuberculosis on livestock farms in south-western Spain. Animals.

[152] Risco D., Salguero F.J., Cerrato R., Gutierrez-Merino J., Lanham-New S., Barquero-Pérez O., Hermoso de Mendoza J., Fernández-Llario P. (2016). Association between vitamin D supplementation and severity of tuberculosis in wild boar and red deer. Res. Vet. Sci..

[153] Naranjo V., Gortazar C., Vicente J., de la Fuente J. (2008). Evidence of the role of European wild boar as a reservoir of *Mycobacterium tuberculosis* complex. Vet. Microbiol..

[154] Vicente J., Höfle U., Garrido J.M., Fernández-DeMera I.G., Juste R., Barral M., Gortazar C. (2006). Wild boar and red deer display high prevalence of tuberculosis-like lesions in Spain. Vet. Res..

[155] Hermoso de Mendoza J., Parra A., Tato A., Alonso J.M., Rey J.M., Peña A., García-Sánchez A., Larrasa J., Teixido J., Manzano G. (2006). Bovine tuberculosis in wild boar (*Sus scrofa*), red deer (*Cervus elaphus*) and cattle (*Bos taurus*) in a Mediterranean ecosystem (1992–2004). Prev. Vet. Med..

[156] Gortázar C., Vicente J., Samper S., Garrido J.M., Fernández-De-Mera I.G., Gavín P., Juste R.A., Martín C., Acevedo P., De La Puente M. (2005). Molecular characterization of *Mycobacterium tuberculosis* complex isolates from wild ungulates in southcentral Spain. Vet. Res..

[157] Artois M., Delahay R., Guberti V., Cheeseman C. (2001). Control of infectious diseases of wildlife in Europe. Vet. J..

[158] Cross P.C., Drewe J., Patrek V., Pearce G., Samuel M.D., Delahay R.J., Delahay R.J., Smith G.C., Hutchings M.R. (2009). Wildlife population structure and parasite transmission: Implications for disease management. Management of Disease in Wild Mammals.

[159] Vicente J., Hofle U., Garrido J.M., Fernández-de-Mera I.G., Acevedo P., Juste R., Barral M., Gortazar C. (2007). Risk factors associated with the prevalence of tuberculosis-like lesions in fenced wild boar and red deer in south central Spain. Vet. Res..

[160] Queirós J., Alves P.C., Vicente J., Gortázar C., de la Fuente J. (2018). Genome-wide associations identify novel candidate loci associated with genetic susceptibility to tuberculosis in wild boar. Sci. Rep..

[161] Acevedo-Whitehouse K., Vicente J., Gortazar C., Höflem U., Fernández-de-Mera I., Amos W. (2005). Genetic resistance to bovine tuberculosis in the Iberian wild boar. Mol. Ecol..

[162] Amos W., Acevedo-Whitehouse K. (2009). A new test for genotype-fitness associations reveals a single microsatellite allele that strongly predicts the nature of tuberculosis in wild boar. Mol. Ecol. Res..

[163] Malo A.F., Coulson T. (2009). Heterozygosity-fitness correlations and associative overdominance: New detection method and proof of principle in the Iberian wild boar. Mol. Ecol..

[164] Barasona J.A., Gortázar C., De La Fuente J., Vicente J. (2019). Host Richness Increases Tuberculosis Disease Risk in Game-Managed Areas. Microorganisms.

[165] Galarza J.A., Sánchez-Fernández B., Fandos P., Soriguer R. (2017). Intensive management and natural genetic variation in red deer (*Cervus elaphus*). J. Hered..

[166] Galarza J., Sánchez-Fernández B., Fandos P., Soriguer R. (2015). The genetic landscape of the Iberian red deer (*Cervus elaphus hispanicus*) after 30 years of big-game hunting in southern Spain. J. Wildl. Manage. Wildl. Monogr..

[167] Herrero-Medrano J.M., Megens H., Groenen M.A., Ramis G., Bosse M., Pérez-Enciso M., Crooijmans R.P.M.A. (2013). Conservation genomic analysis of domestic and wild pig populations from the Iberian Peninsula. BMC Genet..

[168] Pérez-González J., Frantz A.C., Torres-Porras J., Castillo L., Carranza J. (2012). Population structure, habitat features and genetic structure of managed red deer populations. Eur. J. Wildl. Res..

[169] Martínez J.G., Carranza J., Fernández-García J.L., Sánchez-Prieto C.B. (2002). Genetic variation of red deer populations under hunting exploitation in Southwestern Spain. J. Wildl. Manage..

[170] Torres-Porras J., Carranza J., Pérez-González J., Mateos C., Alarcos S. (2014). The tragedy of the commons: Unsustainable population structure of Iberian red deer in hunting estates. Eur. J. Wildl. Res..

[171] Pérez-González J., Carranza J. (2009). Female-biased dispersal under conditions of low male mating competition in a polygynous mammal. Mol. Ecol..

[172] Sánchez-Prieto C.B., Carranza J., Pérez-González J., Alarcos S., Mateos C. (2010). Effects of small barriers on habitat use by red deer: Implications for conservation practices. J. Nat. Conserv..

[173] Pérez-González J., Costa V., Santos P., Slate J., Carranza J., Fernández-Llario P., Zsolnai A., Monteiro N.M., Anton I., Buzgó J. (2014). Males and females contribute unequally to offspring genetic diversity in the polygynandrous mating system of wild boar. PLoS ONE.

[174] Carranza J., Pérez-González J., Mateos C., Fernández-García J.L. (2009). Parents’ genetic dissimilarity and offspring sex in a polygynous mammal. Mol. Ecol..

[175] Pérez-González J., Mateos C., Carranza J. (2009). Polygyny can increase rather than decrease genetic diversity contributed by males relative to females: Evidence from red deer. Mol. Ecol..

[176] Iacolina L., Corlatti L., Buzan E., Safner T., Šprem N. (2019). Hybridization in European ungulates: A review of the current status, causes and consequences. Mammal. Rev..

[177] Randi E. (2005). Management of Wild Ungulate Populations in Italy: Captive-Breeding, Hybridisation and Genetic Consequences of Translocations. Vet. Res. Commun..

[178] Whiteley A.R., Fitzpatrick S.W., Funk W.C., Tallmon D.A. (2015). Genetic rescue to the rescue. Trends Ecol. Evol..

[179] Bell D.A., Robinson Z.L., Funk W.C., Fitzpatrick S.W., Allendorf F.W., Talmon D.A., Whiteley A.R. (2019). The exciting potential and remaining uncertainties of genetic rescue. Trends Ecol. Evol..

[180] Harris K., Zhang Y., Nielsen R. (2019). Genetic rescue and the maintenance of native ancestry. Conserv. Genet..

[181] Carranza J., Martínez J.G., Sánchez-Prieto C., Fernández-García J.L., Sánchez-Fernández B., Álvarez-Álvarez R., Valencia J., Alarcos S. (2003). Game species: Extinctions hidden by census numbers. Anim. Biodivers. Conserv..

[182] Northover A., Lymbery A., Wayne A., Godfrey S., Thompson R. (2018). The hidden consequences of altering host-parasite relationships during fauna translocations. Biol. Conserv..

[183] Sainsbury A.W., Vaughan-Higgins R.J. (2012). Analysing disease risks associated with translocations. Conserv. Biol..

[184] Fernández-de-Mera I.G., Vicente J., Naranjo V., Fierro Y., Garde J.J., de la Fuente J., Gortázar C. (2009). Impact of major histocompatibility complex class II polymorphisms on Iberian red deer parasitism and life history traits. Infect. Genet. Evol..

[185] Frankham R., Ballou J.D., Eldridge M.D., Lacy R.C., Ralls K., Dudash M.R., Fenster C.B. (2011). Predicting the probability of outbreeding depression. Conserv. Biol..

[186] Hedrick P.W., Fredrickson R. (2010). Genetic rescue guidelines with examples from Mexican wolves and Florida panthers. Conserv. Genet..

[187] Casas-Díaz E., Closa-Sebastia F., Peris A., Miño A., Torrentó J., Casanovas R., Marco I., Lavín S., Fernández-Llario P., Serrano E. (2013). Recorded dispersal of wild boar (*Sus scrofa*) in northeast Spain: Implications for disease monitoring programs. Wildl. Biol. Pract..

[188] Albon S.D., Langvatn R. (1992). Plant phenology and the benefits of migration in a temperate ungulate. Oikos.

[189] Pérez-González J., Carranza J. (2011). Female aggregation interacts with population structure to influence the degree of polygyny in red deer. Anim. Behav..

[190] Clutton-Brock T.H., Guinness F.E., Albon S.D. (1982). Red Deer: Behaviour and Ecology of Two Sexes.

[191] Greenwood P.J. (1980). Mating systems philopatry and dispersal in birds and mammals. Anim. Behav..

[192] Pérez-Espona S., Pérez-Barberia F.J., McLeod J.E., Jiggins C.D., Gordon I.J., Pemberton J.M. (2008). Landscape features affect gene flow of Scottish Highland red deer (*Cervus elaphus*). Mol. Ecol..

[193] Foerster K., Coulson T., Sheldon B.C., Pemberton J.M., Clutton-Brock T.H., Kruuk L.E.B. (2007). Sexually antagonistic genetic variation for fitness in red deer. Nature.

[194] Meagher S., Penn D.J., Potts W.K. (2000). Male-male competition magnifies inbreeding depression in wild house mice. Proc. Natl. Scad. Sci. USA.

[195] Pusey A., Wolf M. (1996). Inbreeding avoidance in animals. Trends Ecol. Evol..

[196] Pearse D.E., Anderson E.C. (2009). Multiple paternity increases effective population size. Mol. Ecol..

[197] Sugg D.W., Chesser R.K. (1994). Effective population sizes with multiple paternity. Genetics.

[198] Pérez-González J., Costa V., Santos P., Carranza J., Zsolnai A., Fernández-Llario P., Monteiro N., Anton I., Beja-Pereira A. (2017). Heterozygosity decrease in wild boar mating system—A case of outbreeding avoidance?. J. Zool..

[199] Scandura M., Iacolina L., Apollonio M. (2011). Genetic diversity in the European wild boar *Sus scrofa*: Phylogeography, population structure and wild x domestic hybridization. Mammal. Rev..

[200] Edmands S. (1999). Heterosis and outbreeding depression in interpopulation crosses spanning a wide range of divergence. Evolution.

[201] Barasona J.A., Acevedo P., Diez-Delgado I., Queiros J., Carrasco-García R., Gortazar C., Vicente J. (2016). Tuberculosis-associated death among adult wild boars, Spain, 2009–2014. Emerg. Infect. Dis..

[202] Castillo L., Fernández-Llario P., Mateos C., Carranza J., Benítez-Medina J.M., García-Jiménez W., Bermejo-Martín F., Hermoso de Mendoza J. (2011). Management practices and their association with *Mycobacterium tuberculosis* complex prevalence in red deer populations in Southwestern Spain. Prev. Vet. Med..

[203] Vicente J., Barasona J.A., Acevedo P., Ruiz-Fons J.F., Boadella M., Diez-Delgado I., Beltran-Beck B., González-Barrio D., Queirós J., Montoro V. (2013). Temporal trend of tuberculosis in wild ungulates from Mediterranean Spain. Transbound. Emerg. Dis..

[204] Parra A., García A., Inglis N.F., Tato A., Alonso J.M., Hermoso de Mendoza M., Hermoso de Mendoza J., Larrasa J. (2006). An epidemiological evaluation of *Mycobacterium bovis* infections in wild game animals of the Spanish Mediterranean ecosystem. Res. Vet. Sci..

[205] Aranaz A., de Juan L., Montero N., Sánchez C., Galka M., Delso C., Álvarez J., Romero B., Bezos J., Vela A.I. (2004). Bovine tuberculosis (*Mycobacterium bovis*) in Wildlife in Spain. J. Clin. Microbiol..

[206] Peñuelas J., Lloret F., Montoya R. (2001). Severe drought effects on Mediterranean woody flora in Spain. Forest. Sci..

[207] Olea L., López-Bellido R.J., Poblaciones M.J., Mosquera M.R., Rigueiro A., McAdam J. (2005). Europe types of silvopastoral systems in the Mediterranean area: Dehesa. Silvopastoralism and Sustainable Land Management.

[208] Fernández-Llario P. (2005). The sexual function of wallowing in male wild boar (*Sus scrofa*). J. Ethol..

[209] Cousins D.V. (2001). *Mycobacterium bovis* infection and control in domestic livestock. Rev. Sci. Tech. Off. Int. Epizoot..

[210] Pérez-González J., Barbosa A.M., Carranza J., Torres-Porras J. (2010). Relative effect of food supplementation and natural resources on hind aggregation in a Mediterranean ecosystem. J. Wildl. Manag..

[211] Carranza J., Fernandez-Llario P., Gomendio M. (1996). Correlates of territoriality in rutting red deer. Ethology.

[212] García-Bocanegra I., Pérez de Val B., Arenas-Montes A., Paniagua J., Boadella M., Gortázar C., Arenas A. (2012). Seroprevalence and risk factors associated to *Mycobacterium bovis* in wild artiodactyl species from Southern Spain, 2006–2010. PLoS ONE.

[213] Zanella G., Duvauchelle A., Hars J., Moutou F., Boschiroli M.L., Durand B. (2008). Patterns of lesions of bovine tuberculosis in wild red deer and wild boar. Vet. Rec..

