# Supplementary material for: Host Genetic Diversity and Infectious Diseases. Focus on Wild Boar, Red Deer and Tuberculosis
Source: Animals (Basel). 2021 May 31;11(6):1630. doi: 10.3390/ani11061630 (PMC8229303; doi:10.3390/ani11061630)
Supplement: Supplementary file 1 [file animals-11-01630-s001.zip › animals-1227739-supplementary.pdf]

Host genetic diversity and infectious diseases. Focus on wild boar, red deer, and tuberculosis

Javier Pérez- González[1*], Juan Carranza[2], Remigio Martínez[3], José Manuel Benítez-Medina[3]

Supplementary Material.

Table S1. Number of studies on genetic diversity for ungulate species. Results from a search on the Web of Science with the following search terms: *genetic diversity* and *inbreeding* and *ungulates* (217 studies were obtained). Studies on genetic diversity of ungulate populations published in scientific journals were selected (204 papers). Total: number of studies that explicitly relate genetic diversity to conservation and to disease spread. Cons.: studies that explicitly relate genetic diversity to conservation (papers in which the word 'conservation' appeared in the title, abstract, or the name of the journal; 101 studies, 49.5%). Dise.: studies that explicitly associated genetic diversity with diseases (papers in which the title, abstract, or name of the journal used, at least, one of the following terms: 'disease', 'pathogen', 'parasite', any variation of 'immunity' or the name of any disease; 23 studies, 12.3%). The search was last consulted on 15th April 2021. See Figure 1 and Table S2. Note that the sum of the Total column does not equal 204 because some studies were conducted on several ungulate species.

Ungulate	Total	Cons.	Dise.	Ungulate	Total	Cons.	Dise.
Equus caballus	19	8	1	*Axis porcinus*	1	0	0
Cervus elaphus	16	7	6	*Beatragus hunteri*	1	1	0
Bison bonasus	10	7	1	*Bos javanicus*	1	1	0
Oryx leucoryx	9	4	1	*Camelus dromedarius*	1	0	0
Ovis aries	9	4	1	*Camelus ferus*	1	1	0
Ovis canadensis	9	2	0	*Capra aegagrus*	1	0	0
Bos taurus	7	2	1	*Capra pyrenaica*	1	0	0
Cervus nippon	7	1	0	*Cervus albirostris*	1	1	0
Sus scrofa	6	3	0	*Cervus unicolor*	1	1	0
Bison bison	6	5	1	*Choeropsis liberiensis*	1	0	0
Diceros bicornis	6	5	0	*Dama dama*	1	1	0
Cervus eldii	5	4	0	*Gazella gazelle*	1	1	0
Elaphurus davidianus	5	3	1	*Gazelle dorca*	1	0	0
Rangifer tarandus	5	3	0	*Giraffa camelopardalis*	1	0	0
Capreolus capreolus	4	0	1	*Giraffa giraffa*	1	1	0
Equus hemious	4	2	0	*Hippocamelus bisulcus*	1	1	0
Gazella cuvieri	4	1	0	*Hippotragus niger*	1	1	0
Oreamnos americanus	4	2	1	*Hydropotes inermis*	1	0	0
Capra hircus	3	0	0	*Moschus moschiferus*	1	0	0

Ceratotherium simum	3	3	0	*Muntiacus crinifrons*	1	0	0
Equus zebra	3	2	3	*Naemorhedus baileyi*	1	1	0
Gazella spekei	3	1	0	*Naemorhedus griseus*	1	1	0
Moschus berezovskii	3	2	0	*Nanger soemmerringii*	1	0	0
Nanger dama	3	1	0	*Odocoileus hemionus*	1	1	1
Odocoileus virginianus	3	2	0	*Okapia johnstoni*	1	1	0
Ovibos moschatus	3	0	0	*Ovis orientalis*	1	0	0
Antidorcas marsupialis	2	0	1	*Porcula salvania*	1	1	0
Bos mutus	2	1	0	*Rhinoceros unicornis*	1	1	0
Bubalus bubalis	2	0	1	*Rucervus duvaucelii*	1	0	0
Capra ibex	2	1	0	*Rucervus eldii*	1	1	0
Cervus canadensis	2	1	0	*Rusa timorensis*	1	0	0
Equus quagga	2	1	1	*Saiga tatarica*	1	0	0
Lama guanicoe	2	1	0	*Sus cebrifons*	1	1	0
Oryx dammah	2	2	0	*Tapirus bairdii*	1	1	0
Rupicapra rupicapra	2	2	0	*Tapirus terrestris*	1	1	0
Syncerus caffer	2	1	1	*Trafelaphus imberbis*	1	0	0
Taurotragus derbianus	2	2	0	*Tragelaphus angasii*	1	0	0
Vicugna vicugna	2	1	0	*Tragelaphus imberbis*	1	0	0
Addax nasomaculatus	1	1	0	*Tragelaphus sylvaticus*	1	0	0
Alces alces	1	0	0	*Trofelaphus isaaci*	1	0	0

Table S2. Number of studies in scientific journals (ungulates). Number of studies published in scientific journals for the search on the Web of Science with the following search terms: *genetic diversity* and *inbreeding* and *ungulates*. See Figure 1 and Table S1.

Journal	Studies	Journal	Studies
Conservation Genetics	19	Biology Letters	1
Plos One	11	Biology of Reproduction	1
Journal of Heredity	9	BMC Genetics	1
Molecular Ecology	8	Chinese Science Bulletin	1
Zoo Biology	8	Contributions to Zoology	1
Journal of Wildlife Management	7	Cryobiology	1
Biological Conservation	6	Current Zoology	1
Animal Conservation	5	Frontiers in Ecology & Evolution	1
Biochemical Systematics and Ecology	5	Gene	1
Ecology and Evolution	5	Genes & Genetic Systems	1
Journal of Zoology	5	Genética	1
Mammalian Biology	5	Genetics	1
Animal Genetics	4	Genetika	1
Biological Journal of the Linnean Society	4	Genome Biology and Evolution	1
Conservation Biology	4	Genomics	1
Evolutionary Applications	4	Global Change Biology	1
Indian Journal of Animal Sciences	4	Iceland Agricultural Sciences	1

Acta Theriologica	3	Infection Genetics and Evolution	1
BMC Genomics	3	International Journal of Genomics	1
Conservation Genetics Resources	3	Italian Journal of Animal Science	1
European Journal of Wildlife Research	3	Journal of Animal and Plant Sciences	1
Genetics and Molecular Research	3	Journal of Applied Genetics	1
Molecular Ecology Resources	3	Journal of Biogeography	1
Russian Journal of Genetics	3	Journal of Experimental Zoology	1
Biochemical Genetics	2	Journal of Fish and Wildlife Management	1
BMC Evolutionary Biology	2	Journal of Mammalogy	1
Canadian Journal of Zoology	2	Journal of the South African Veterinary Association	1
Evolution	2	Journal of Zoo and Aquarium Research	1
Folia Zoologica	2	Mammal Study	1
Global Ecology and Conservation	2	Mitochondrial DNA	1
Heredity	2	Mitochondrial DNA part A	1
Journal of Animal Breeding and Genetics	2	Oecologia	1
Mammal Review	2	Pachyderm	1
Scientific Reports	2	Plos Genetics	1
Wildlife Biology	2	Reproduction in Domestic Animals	1
African Journal of Biotechnology	1	Revista Brasileira de Zootecnia	1
African Journal of Ecology	1	Revista de Investigaciones Veterinarias del Perú	1
Animal Behaviour	1	South African Journal of Wildlife Research	1
Animal Biodiversity and Conservation	1	South American Camelids Research	1
Applied Animal Behaviour Science	1	Western North American Naturalists	1
Australian Journal of Zoology	1	Zoological Journal of the Lineean Society	1
Belgian Journal of Zoology	1		

Table S3. Number of studies in scientific journals (wild boar). Number of studies published in scientific journals for the search on the Web of Science with the following search terms: *wild boar* and *Sus scrofa* and *tuberculosis*. See Figure 2 and Table 1.

Journal	Studies	Journal	Studies
Transboundary and Emerging Diseases	20	Biomed Research International	1
Preventive Veterinary Medicine	19	Comp. Immun. Migrobiol. Infect. Dis.	1
Plos One	16	Ecological Indicators	1
European Journal of Wildlife Research	11	Environmental Toxicology and Chemistry	1
Veterinary Microbiology	11	Expert Review of Vaccines	1
Journal of Wildlife Diseases	9	Fems Immunology and Medical Microbiology	1
BMC Veterinary Research	6	Food Control	1
Frontiers in Veterinary Science	6	Foodborne Pathogens and Disease	1
Journal of Clinical Microbiology	6	Frontiers in Microbiology	1
Pathogens	6	Infection Genetics and Evolution	1
Research in Veterinary Science	6	Int. J. System. Evol. Mycrobiol.	1
Veterinary Record	6	Journal of Animal Ecology	1
Veterinarni Medicina	5	Journal of Fish Diseases	1

Veterinary Research	5	Journal of Food Protection	1
Veterinary Journal	4	Journal of Veterinary Diagnostic Investigation	1
Emerging Infectious Diseases	3	Journal of Veterinary Science	1
Epidemiology and Infection	3	Journal of Zoology	1
Mammal Review	3	Medical Hypotheses	1
Molecular Ecology	3	Medycyna Weterynaryjna-Vet. Med.-Sci. Prac.	1
Scientific Reports	3	Microbial Pathogens	1
Vaccine	3	Microorganisms	1
Veterinary Immunology and Immunopath.	3	Molecular Biology Reports	1
Clinical and Vaccine Immunology	2	Monatshefte fur Veterinarmedizin	1
Ecohealth	2	New Zeland Veterinary Journal	1
Journal of Veterinary Medicine	2	One Health	1
Magyar Allatorvosok Lapja	2	Philosophical Transactions of the Royal Society	1
Tieraerzliche Umschau	2	Plos Neglected Tropical Diseases	1
Tuberculosis	2	Polish Journal of Veterinary Sciences	1
Veterinarski Arhiv	2	Proteomics	1
Veterinary Medicine International	2	Sensors	1
Acta Tropica	1	Slovenian Veterinary Research	1
Acta Veterinaria Hungarica	1	The Veterinary Record	1
Acta Veterinaria Scandinavica	1	Tropical Animal Health and Production	1
Adv. Microbiol., Infect. Dis, Pub. Health	1	Veterinary Medicine	1
American Naturalist	1	Wiener Tierarztliche Monatsschrift	1
Animal Genetics	1	Wildlife Research	1
Animals	1	Zoonoses and Public Health	1

Table S4. Number of studies in scientific journals (red deer). Number of studies published in scientific journals for the search on the Web of Science with the following search terms: *red deer* and *Cervus elaphus* and *tuberculosis*. See Figure 2 and Table 1.

Journal	Studies	Journal	Studies
New Zeland Veterinary Journal	19	Vaccine	2
Veterinary Record	16	Veterinary Research Communications	2
Journal of Wildlife Diseases	15	Wiener Tierarztliche Monatsschrift	2
Preventive Veterninary Medicine	14	Acta Veterinaria Hungarica	1
Veterinary Microbiology	10	Animal Production Science	1
European Journal of Wildlife Research	8	Animals	1
Plos One	7	Archivos de Medicina Veterinaria	1
Transboundary and Emerging Diseases	7	Biological Journal of the Linnean Society	1
Veterinary Journal	6	Canadian Journal of Zoology	1
Research in Veterinary Science	5	Clini. Diag. Lab. Immun.	1
Veterinarni Medicina	5	Food Control	1
BMC Veterinary Research	4	Food Microbiology	1
Clinical and Vaccine Immunology	4	Immunology and Cell Biology	1
Journal of Clinical Microbiology	4	ISRN Veterinary Science	1

Journal of Veterinary Diagnostic Investigation	4	Journal of Applied Microbiology	1
Veterinary Immunology and Immunopathology	4	Journal of The Royal Society of New Zeland	1
Veterinary Research	4	Journal of Veterinary Science	1
American Journal of Veterinary Reserach	3	Journal of Wildlife Management	1
Canadian Veterinary Journal	3	Lancet	1
Epidemilogy and Infection	3	Magyar Alltorvosok Lapja	1
Frontiers in Veterinary Science	3	Mammal Review	1
Infection and Immunity	3	Microorganisms	1
Berliner Und Munchener Tierarztliche Wochens.	2	Oecologia	1
Canadian Journal of Veterinary Research	2	Rev. Scient. Tech. Office Intern. Epiz.	1
Cattle Practice	2	Scandinavian Journal of Immunology	1
Comp. Immun. Migrobiol. Infect. Dis.	2	Scientific Reports	1
Developmental and Comparative Immunology	2	The Veterinary Record Volumen	1
Emerging Infectious Diseases	2	Tijdschrift voor Diergeneeskunde	1
Infection Genetics and Evolution	2	Trends in Game Meat Hygiene	1
Journal of Comparative Pathology	2	Tropical Animal Health and Production	1
J. Am. Vet. Med. Assoc.	2	Tubercle and Lung Disease	1
Journal of Veterinary Medical Science	2	Veterinary Pathology	1
Pathogens	2	Wildlife Society Bulletin	1
Pesquisa Veterinaria Brasileira	2	Zoonoses and Public Health	1
Tuberculosis	2		
